# A First Study of Inheritance of Epilepsy

**Published:** 1912-02

**Authors:** 


A FIRST STUDY OF INHERITANCE OF EPILEPSY, Charles B. Davenport and David F. Weeks, M.D., *Journal of Nervous and Mental Disease,* November, 1911.

This is an analysis of the data obtained in 177 pedigrees of the patients at the New Jersey Village for Epileptics at Skillman, N. J. The pedigrees were obtained by field-workers who visited the homes of the relatives and in that way were able to obtain data which would be otherwise impossible, as it is well known how difficult it is to obtain data on blank forms sent out by an institution.

These two experienced workers in Eugenics and in Epilepsy have employed the Mendelian method in analyzing the material obtained by their field-workers. This method assumes that the inheritance of any character is not from the parents, grandparents, etc., but from the germ-plasm out of which every fraternity and its parents and other relatives have risen, and accordingly, when a unit character, upon which normal development depends, is absent from the germ-plasm that character cannot be transmitted to the offspring.

The genealogic charts and tables accompanying the article show in a most instructive manner the preponderance of epilepsy, feeble-mindedness, etc., in these families. In one instance there were 35 F x F (feeble-minded with feeble-minded) matings, which yielded 142 known offsprings—all feeble-minded.

As a result of these analyses the authors conclude that when both parents are epileptic, both feeble-minded, or one epileptic and the other feeble-minded all the offspring will be either epileptic or feeble-minded, and the proportion of epileptics in any fraternity increases with that in the parentage.

When epilepsy occurs in the progeny out of matings between "tainted" and "normal" persons they conclude that the normal parents must belong to defective strains, as it would seem to be rare that a person of mentally strong strain, even if mated to a "tainted" person, should have an epileptic child. The normal parent of an epileptic usually has some defective germ cells.

The writers advance the hypothesis that, apart possibly from traumatic causes, epilepsy rarely, if ever, arises from strains devoid of defective germ plasm.

From a sociological standpoint the data given in this paper are of great value, and as the evidence accumulates that the reproduction of these defective classes should be limited it may open the way to legislative enactments restricting the marriage of such individuals.

The authors of the paper state, that, "If our data should hold generally for strains with epileptic members we could conclude that, if no change in mating and fecundity occur, the number of epileptics and feeble-minded in the state of New Jersey will be relatively double what it is now in 1940, and relatively four times as common in 1970. Thus if the present proportion is 1 to 500 it would be 1 to 125 in 1970."

As measures to stop this increase they recommend as the most effective, and violating least the social ideals of our time, the segregation during the entire reproductive period (say from 15 to 45 years of age) of epileptics of both sexes.

